# Supplementary material for: Spatiotemporal orchestration of calcium-cAMP oscillations on AKAP/AC nanodomains is governed by an incoherent feedforward loop
Source: PLoS Comput Biol. 2024 Oct 31;20(10):e1012564. doi: 10.1371/journal.pcbi.1012564 (PMC11556706; doi:10.1371/journal.pcbi.1012564)
Supplement: S1 Table — (PDF) [file pcbi.1012564.s001.pdf]

Abbreviation	Explanation
**Species in the cytosol**	
Ca^{2+}	Calcium
CaM	Calmodulin
Ca_2CaM	Ca^{2+}-bound CaM with two Ca^{2+} atoms
Ca_3CaM	Ca^{2+}-bound CaM with three Ca^{2+} atoms
Ca_4CaM	Ca^{2+}-bound CaM with four Ca^{2+} atoms
PDE	Cyclic nucleotide phosphodiesterase (inactive form)
CaM · PDE	Complex of CaM and PDE
PDE*	Activated PDE
cAMP	Cyclic adenosine monophosphate
R_2	Protein kinase A with only two regulatory subunits
R_2C	Protein kinase A with one catalytic subunit and two regulatory subunits
R_2C_2	Protein kinase A with two catalytic subunits and two regulatory subunits
PKA	Active protein kinase A
**Species on the membrane**	
V	Membrane voltage
w	K^+ channel open probability
AC	Adenylyl cyclase (inactive form)
CaM · AC	Complex of CaM and AC
AC*	Activated AC
AKAP	A-kinase anchoring protein
AKAP-R_2C_2	Complex of AKAP and R_2C_2
AKAP-R_2C	Complex of AKAP and R_2C
AKAP-R_2	Complex of AKAP and R_2
