# Supplementary material for: Spatiotemporal orchestration of calcium-cAMP oscillations on AKAP/AC nanodomains is governed by an incoherent feedforward loop
Source: PLoS Comput Biol. 2024 Oct 31;20(10):e1012564. doi: 10.1371/journal.pcbi.1012564 (PMC11556706; doi:10.1371/journal.pcbi.1012564)
Supplement: S2 Table — (PDF) [file pcbi.1012564.s002.pdf]

#	Reaction	Reaction flux
v1	$Ca^{2+} + CaM \leftrightarrow Ca_2CaM$	$j_1 = k_f[\mathrm{Ca}^{2+}][\mathrm{CaM}] - k_r[\mathrm{Ca_2CaM}]$
v2	$Ca^{2+} + Ca_2CaM \leftrightarrow Ca_3CaM$	$j_2 = k_f[\mathrm{Ca}^{2+}][\mathrm{Ca_2CaM}] - k_r[\mathrm{Ca_3CaM}]$
v3	$Ca^{2+} + Ca_3CaM \leftrightarrow Ca_4CaM$	$j_3 = k_f[\mathrm{Ca}^{2+}][\mathrm{Ca_3CaM}] - k_r[\mathrm{Ca_4CaM}]$
v4	$AC + Ca_2CaM \leftrightarrow CaM \cdot AC$	$j_4 = k_f[\mathrm{AC}][\mathrm{Ca_2CaM}] - k_r[\mathrm{CaM \cdot AC}]$
v5	$CaM \cdot AC + 2\,Ca^{2+} \leftrightarrow AC^*$	$j_5 = K_{cat}\frac{[\mathrm{Ca}^{2+}][\mathrm{CaM \cdot AC}]}{[\mathrm{Ca}^{2+}]+K_m} - k_r[\mathrm{AC}^*]$
v6	$PDE + Ca_2CaM \leftrightarrow CaM \cdot PDE$	$j_6 = k_f[\mathrm{PDE}][\mathrm{Ca_2CaM}] - k_r[\mathrm{CaM \cdot PDE}]$
v7	$CaM \cdot PDE + 2\,Ca^{2+} \leftrightarrow PDE^*$	$j_7 = K_{cat}\frac{[\mathrm{Ca}^{2+}][\mathrm{CaM \cdot PDE}]}{[\mathrm{Ca}^{2+}]+K_m} - k_r[\mathrm{AC}^*]$
v8	$PDE + Ca_4CaM \leftrightarrow PDE^*$	$j_8 = k_f[\mathrm{PDE}][\mathrm{Ca_4CaM}] - k_r[\mathrm{PDE}^*]$
v9	$\rightarrow cAMP$	$j_9 = k_{base}([\mathrm{CaM \cdot AC}] + AC_{ind} + [\mathrm{AC}^*]) + k_{act}[\mathrm{AC}^*]$
v10	$cAMP\rightarrow$	$j_{10} = k_{base}[\mathrm{PDE}]\frac{[\mathrm{cAMP}]}{[\mathrm{cAMP}]+K_m}$
v11	$cAMP\rightarrow$	$j_{11} = k_{base}[\mathrm{CaM \cdot PDE}]\frac{[\mathrm{cAMP}]}{[\mathrm{cAMP}]+K_m}$
v12	$cAMP\rightarrow$	$j_{12} = k_{act}[\mathrm{PDE}^*]\frac{[\mathrm{cAMP}]}{[\mathrm{cAMP}]+K_m}$
v13	$cAMP\rightarrow$	$j_{13} = k_{base}\frac{[\mathrm{cAMP}]}{[\mathrm{cAMP}]+K_m}$
v14	$2\,cAMP + R_2C_2 \leftrightarrow R_2C + PKA$	$j_{14} = k_f[\mathrm{cAMP}]^2[\mathrm{R_2C_2}] - k_r[\mathrm{R_2C}][\mathrm{PKA}]$
v15	$2\,cAMP + R_2C \leftrightarrow R_2 + PKA$	$j_{15} = k_f[\mathrm{cAMP}]^2[\mathrm{R_2C}] - k_r[\mathrm{R_2}][\mathrm{PKA}]$
v16	$AKAP + R_2C_2 \leftrightarrow AKAP\text{-}R_2C_2$	$j_{16} = k_f[\mathrm{AKAP}][\mathrm{R_2C_2}] - k_r[\mathrm{AKAP\text{-}R_2C_2}]$
v17	$AKAP + R_2C \leftrightarrow AKAP\text{-}R_2C$	$j_{17} = k_f[\mathrm{AKAP}][\mathrm{R_2C}] - k_r[\mathrm{AKAP\text{-}R_2C}]$
v18	$AKAP + R_2 \leftrightarrow AKAP\text{-}R_2$	$j_{18} = k_f[\mathrm{AKAP}][\mathrm{R_2}] - \mathrm{k}_r[\mathrm{AKAP\text{-}R_2}]$
v19	$2\,cAMP + AKAP\text{-}R_2C_2 \leftrightarrow$ $AKAP\text{-}R_2C + PKA$	$j_{19} = k_f[\mathrm{cAMP}]^2[\mathrm{AKAP\text{-}R_2C_2}] - k_r[\mathrm{AKAP\text{-}R_2C}][\mathrm{PKA}]$
v20	$2\,cAMP + AKAP\text{-}R_2C \leftrightarrow$ $AKAP\text{-}R_2 + PKA$	$j_{20} = k_f[\mathrm{cAMP}]^2[\mathrm{AKAP\text{-}R_2C}] - k_r[\mathrm{AKAP\text{-}R_2}][\mathrm{PKA}]$
v23	$\rightarrow Ca^{2+}$	$j_{23} = Ck_{IP3R}[\mathrm{PKA}]\frac{A[\mathrm{Ca}^{2+}](\mathrm{Ca_{stores}}-[\mathrm{Ca}^{2+}])}{1+A[\mathrm{Ca}^{2+}]+B[\mathrm{Ca}^{2+}]^2} - \frac{V_s[\mathrm{Ca}^{2+}]^2}{K_S^2+[\mathrm{Ca}^{2+}]^2}$
