# Supplementary material for: Spatiotemporal orchestration of calcium-cAMP oscillations on AKAP/AC nanodomains is governed by an incoherent feedforward loop
Source: PLoS Comput Biol. 2024 Oct 31;20(10):e1012564. doi: 10.1371/journal.pcbi.1012564 (PMC11556706; doi:10.1371/journal.pcbi.1012564)
Supplement: S3 Table — (PDF) [file pcbi.1012564.s003.pdf]

#	Current or open probability	Expression
Current	Ca^{2+} gated K^+ channel current (v21)	$I_{KCa} = g_{KCa}(V - E_{KCa})\frac{[\mathrm{Ca}^{2+}]}{[\mathrm{Ca}^{2+}]+K_{KCa}}$
	Ca^{2+} channel current	$I_{Ca} = g_{Ca}(V - E_{Ca})\frac{1+tanh\left(\frac{V-v_1}{v_2}\right)}{2}$
	Leak channel current	$I_L = g_L(V - E_L)$
	K^+ channel current	$I_K = g_K(V - E_K)w$
K^+ channel	Time constant for K^+ channel open probability	$\tau = \frac{1}{cosh\left(\frac{V-v_3}{2v_4}\right)}$
	Steady state fraction of K^+ channel	$w_\infty = \frac{1+tanh\left(\frac{V-v_3}{v_4}\right)}{2}$
	K^+ channel open probability	$\frac{dw}{dt} = \phi\frac{w_\infty - w}{\tau}$
v22	Ca^{2+} flux across plasma membrane	$j_{22} = \Big(-\alpha I_{Ca} - v_{LPM}[\mathrm{Ca}^{2+}]\Big)(1 + k_{PKAV}[\mathrm{PKA}])$
