# Supplementary material for: Spatiotemporal orchestration of calcium-cAMP oscillations on AKAP/AC nanodomains is governed by an incoherent feedforward loop
Source: PLoS Comput Biol. 2024 Oct 31;20(10):e1012564. doi: 10.1371/journal.pcbi.1012564 (PMC11556706; doi:10.1371/journal.pcbi.1012564)
Supplement: S4 Table — (PDF) [file pcbi.1012564.s004.pdf]

Kinetic parameters	Definitions	Values
C_m	Capacitance of cell membrane	5.3 pF [46]
k_f in j_1	Forward rate	3.6 $s^{-1} \cdot \mu M^{-1}$ [49]
k_r in j_1	Backward rate	8 s^{-1} [49]
k_f in j_2	Forward rate	11 $s^{-1} \cdot \mu M^{-1}$ [49]
k_r in j_2	Backward rate	195 s^{-1} [49]
k_f in j_3	Forward rate	59 $s^{-1} \cdot \mu M^{-1}$[49]
k_r in j_3	Backward rate	500 s^{-1} [49]
k_f in j_4	Forward rate	6.8 $s^{-1} \cdot \mu M^{-1}$ [Constraint]
k_r in j_4	Backward rate	10 s^{-1} [Constraint]
K_{cat} in j_5	Forward rate	90 s^{-1}[Constraint]
K_m in j_5	Activation constant	1 μM [Constraint]
k_r in j_5	Backward rate	10 s^{-1} [Constraint]
k_f in j_6	Forward rate	0.25 $s^{-1} \cdot \mu M^{-1}$[Constraint]
k_r in j_6	Backward rate	1 s^{-1} [Constraint]
K_{cat} in j_7	Forward rate	60 s^{-1} [Constraint]
K_m in j_7	Half-maximal effective concentration	1 μM [Constraint]
k_r in j_7	Backward rate	1 s^{-1} [Constraint]
k_f in j_8	Forward rate	0.25 $s^{-1} \cdot \mu M^{-1}$[Constraint]
k_r in j_8	Backward rate	0.1 s^{-1} [Constraint]
k_{base} in j_9	Basal activation rate	0.2 s^{-1} [Constraint]
k_{act} in j_9	Activation rate caused by active AC	23.55 s^{-1} [Constraint]
AC_{ind} in j_9	Basal AC level	3E-8 mol/m^2 [Constraint]
k_{base} in j_{10}	Basal activation rate	0.6 s^{-1} [Constraint]
K_m in j_{10}	Half-maximal effective concentration	0.6 μM [Constraint]
k_{base} in j_{11}	Basal activation rate	0.6 s^{-1} [Constraint]
K_m in j_{11}	Half-maximal effective concentration	0.6 μM [Constraint]
k_{act} in j_{12}	Activation rate for active AC	720 s^{-1} [Constraint]
K_m in j_{12}	Half-maximal effective concentration	0.6 μM [Constraint]

k_{base} in j_{13}	Basal activation rate	0.25 μM $\cdot$ s^{-1} [Constraint]
K_m in j_{13}	Half-maximal effective concentration	1.4 μM [Constraint]
k_f in j_{14}	Forward rate	33.33 $s^{-1} \cdot$ μM^{-2} [Estimated]
k_r in j_{14}	Backward rate	20 $s^{-1} \cdot$ μM^{-1} [Estimated]
k_f in j_{15}	Forward rate	33.33 $s^{-1} \cdot$ μM^{-2} [Estimated]
k_r in j_{15}	Backward rate	20 $s^{-1} \cdot$ μM^{-1} [Estimated]
k_f in j_{16}	Forward rate	1 $s^{-1} \cdot$ μM^{-1} [Estimated]
k_r in j_{16}	Backward rate	0.1 s^{-1} [Estimated]
k_f in j_{17}	Forward rate	1 $s^{-1} \cdot$ μM^{-1} [Estimated]
k_r in j_{17}	Backward rate	0.1 s^{-1} [Estimated]
k_f in j_{18}	Forward rate	1 $s^{-1} \cdot$ μM^{-1} [Estimated]
k_r in j_{18}	Backward rate	0.1 s^{-1} [Estimated]
k_f in j_{19}	Forward rate	33.33 $s^{-1} \cdot$ μM^{-2} [Estimated]
k_r in j_{19}	Backward rate	20 $s^{-1} \cdot$ μM^{-1} [Estimated]
k_f in j_{20}	Forward rate	33.33 $s^{-1} \cdot$ μM^{-2} [Estimated]
k_r in j_{20}	Backward rate	20 $s^{-1} \cdot$ μM^{-2} [Estimated]
g_{KCa} in I_{KCa}	Conductance of Ca^{2+} gated K^+ channel	2000 pS [46]
E_{KCa} in I_{KCa}	Reversal potential of Ca^{2+} gated K^+ channel	-75 mV [46]
K_{KCa} in I_{KCa}	Half-maximal effective concentration	5 μM [46]
g_{Ca} in I_{Ca}	Conductance of Ca^{2+} channel	600 pS [46]
E_{Ca} in I_{Ca}	Reversal potential of Ca^{2+} channel	100 mV [46]
v_1 in I_{Ca}	Potential when the steady-state fraction of open Ca^{2+} channel is 0.5	-20 mV [46]
v_2 in I_{Ca}	Normalization factor	24 mV [46]
g_L in I_L	Conductance of leak channel	150 pS [46]
E_L in I_L	Reversal potential of leak channel	-75 mV [46]
g_K in I_K	Conductance of K^+ channel	260 pS [46]
E_K in I_K	Reversal potential of K^+ channel	-75 mV [46]
ϕ	Factor controlling relative time scales of V and w	35 s^{-1} [46]
v_3	Potential when the steady-state fraction of open	-16 mV [46]

	K^+ channel is 0.5	
v_4	Normalization factor	11.2 mV [46]
I_{Ca} in j_{22}	Ca^{2+} channel current	Defined in S3 Table
α in j_{22}	Factor converting current to $\mu M \cdot s^{-1}$	4.15E-5 $mol \cdot m^{-2} \cdot A^{-2} \cdot s^{-1}$ [46]*
v_{LPM} in j_{22}	Rate constant for Ca^{2+} leak through membrane	7.5E-4 $m \cdot s^{-1}$ [46]*
k_{PKAV} in j_{22}	Coefficient of modulation of Ca^{2+} fluxes by PKA	100 μM^{-1} [Constraint]
K_s in j_{23}	Half-maximal effective concentration	10 μM [46]
Ca_{stores} in j_{23}	Total Ca^{2+} in the cytosol	1.56 μM [46]
V_s in j_{23}	Activation rate	0.1 $\mu M \cdot s^{-1}$ [46]
k_{IP3R} in j_{23}	Activation rate caused by IP3 receptor	0.05 $\mu M \cdot s^{-1}$ [46]*
A in j_{23}	Reaction rate constant	0.2869 μM^{-1} [46]
B in j_{23}	Reaction rate constant	2.869 μM^{-2} [46]
C in j_{23}	Reaction rate constant	0.2133 [46]
