# Supplementary material for: Spatiotemporal orchestration of calcium-cAMP oscillations on AKAP/AC nanodomains is governed by an incoherent feedforward loop
Source: PLoS Comput Biol. 2024 Oct 31;20(10):e1012564. doi: 10.1371/journal.pcbi.1012564 (PMC11556706; doi:10.1371/journal.pcbi.1012564)
Supplement: S5 Table — (PDF) [file pcbi.1012564.s005.pdf]

Kinetic parameters	Definitions	Values
$D_{\mathrm{Ca}^{2+}}$	Diffusion coefficient of Ca^{2+}	100 μm^2 s^{-1}
D_{CaM}	Diffusion coefficient of CaM	10 μm^2 s^{-1}
$D_{\mathrm{Ca_2CaM}}$	Diffusion coefficient of CaM_2CaM	10 μm^2 s^{-1}
$D_{\mathrm{Ca_3CaM}}$	Diffusion coefficient of CaM_3CaM	10 μm^2 s^{-1}
$D_{\mathrm{Ca_4CaM}}$	Diffusion coefficient of CaM_4CaM	10 μm^2 s^{-1}
D_{PDE}	Diffusion coefficient of PDE	10 μm^2 s^{-1}
$D_{\mathrm{CaM \cdot PDE}}$	Diffusion coefficient of CaM · PDE	10 μm^2 s^{-1}
$D_{\mathrm{PDE^*}}$	Diffusion coefficient of PDE*	10 μm^2 s^{-1}
D_{cAMP}	Diffusion coefficient of cAMP	60 μm^2 s^{-1}
$D_{\mathrm{R_2}}$	Diffusion coefficient of R_2	10 μm^2 s^{-1}
$D_{\mathrm{R_2C}}$	Diffusion coefficient of R_2C	10 μm^2 s^{-1}
$D_{\mathrm{R_2C_2}}$	Diffusion coefficient of R_2C_2	10 μm^2 s^{-1}
D_{PKA}	Diffusion coefficient of PKA	0.01 μm^2 s^{-1}
D_V	Diffusion coefficient of the membrane voltage	1 μm^2 s^{-1}
