# Supplementary material for: Spatiotemporal orchestration of calcium-cAMP oscillations on AKAP/AC nanodomains is governed by an incoherent feedforward loop
Source: PLoS Comput Biol. 2024 Oct 31;20(10):e1012564. doi: 10.1371/journal.pcbi.1012564 (PMC11556706; doi:10.1371/journal.pcbi.1012564)
Supplement: S6 Table — (PDF) [file pcbi.1012564.s006.pdf]

Kinetic parameters	Definitions	Values
$[Ca^{2+}]_0$	Initial condition of $[Ca^{2+}]$	0.001 μM
$[CaM]_0$	Initial condition of [CaM]	2.9 μM
$[Ca_2CaM]_0$	Initial condition of $[CaM_2CaM]$	0.1 μM
$[Ca_3CaM]_0$	Initial condition of $[CaM_3CaM]$	4E-3 μM
$[Ca_4CaM]_0$	Initial condition of $[CaM_4CaM]$	1E-2 μM
$[PDE]_0$	Initial condition of [PDE]	0.9 μM
$[CaM \cdot PDE]_0$	Initial condition of [CaM · PDE]	1E-3 μM
$[PDE^*]_0$	Initial condition of [PDE*]	1E-3 μM
$[cAMP]_0$	Initial condition of [cAMP]	4E-6 μM
$[R_2]_0$	Initial condition of $[R_2]$	0.04 μM
$[R_2C]_0$	Initial condition of $[R_2C]$	0
$[R_2C_2]_0$	Initial condition of $[R_2C_2]$	0.2 μM
$[PKA]_0$	Initial condition of [PKA]	0.05 μM
V_0	Initial condition of the membrane voltage V	-60 mV
w_0	Initial condition of the K^+ channel open probability w	0
$[AC]_0$	Initial condition of [AC]	Specified in the main text
$[CaM \cdot AC]_0$	Initial condition of [CaM · AC]	0
$[AC^*]_0$	Initial condition of [AC*]	0
$[AKAP]_0$	Initial condition of [AKAP]	Specified in the main text
$[AKAP\text{-}R_2]_0$	Initial condition of $[AKAP\text{-}R_2]$	0
$[AKAP\text{-}R_2C]_0$	Initial condition of $[AKAP\text{-}R_2C]$	0
$[AKAP\text{-}R_2C_2]_0$	Initial condition of $[AKAP\text{-}R_2C_2]$	0
$\frac{\partial X}{\partial t}\|_{t=0}$	The derivative of X w.r.t time at t= 0 ($X = [Ca^{2+}]$, [CaM], ..., $[AKAP\text{-}R_2C_2]$)	0
