# Supplementary material for: Spatiotemporal orchestration of calcium-cAMP oscillations on AKAP/AC nanodomains is governed by an incoherent feedforward loop
Source: PLoS Comput Biol. 2024 Oct 31;20(10):e1012564. doi: 10.1371/journal.pcbi.1012564 (PMC11556706; doi:10.1371/journal.pcbi.1012564)
Supplement: S7 Table — (PDF) [file pcbi.1012564.s007.pdf]

Kinetic parameters	Values
b	0.1 s^{-1}
m_1	0.04 s^{-1}
m_2	1 s^{-1}
β	0.05 s^{-1}
μ	0.07 s^{-1}
D_r	1.25×10^{-3} µm^2 s^{-1}
D_s	2.5×10^{-5} µm^2 s^{-1}
$\overline{r}$	0.05
$\overline{s}$	0.05
