# Supplementary material for: Spatiotemporal orchestration of calcium-cAMP oscillations on AKAP/AC nanodomains is governed by an incoherent feedforward loop
Source: PLoS Comput Biol. 2024 Oct 31;20(10):e1012564. doi: 10.1371/journal.pcbi.1012564 (PMC11556706; doi:10.1371/journal.pcbi.1012564)
Supplement: Attachment — Submitted filename: Review_of_PCOMPBIOL_D_24_00398.pdf [file pcbi.1012564.s014.pdf]

Review of PCOMPBIOL-D-24-00398: Spatiotemporal orchestration of Ca^{2+}-cAMP oscillations on AKAP/AC nanodomains is governed by an incoherent feedforward loop

Andrew L. Krause, Durham University, UK*

The authors explore models of Ca^{2+}-cAMP oscillations and how they are influenced by the presence of AKAP/AC nanodomains which form on cell membranes. They argue that these oscillations play a pivotal role in many diseases and developmental/physiological function, and that the impact of membrane nanodomains on the local profiles of such oscillations can have important effects. They use a range of simple and quite complicated models of reaction-diffusion type to explore in-phase and perfectly out-of-phase oscillations mediated by being inside and outside, respectively, of these nanodomains. They show that such dynamics are qualitatively robust to biophysical details, such as compartment size and geometry/spacing of nanodomains. They also postulate a simple model of nanodomain formation via a Turing reaction-diffusion mechanism, though caveat this as only one plausible mechanism for such pattern formation. Importantly, they highlight throughout a simplistic motif of a feedforward incoherent feedback loop that can help explain the phase of these Ca^{2+}-cAMP oscillations.

Overall I find the paper interesting, and broadly well-written. Below are a few rather minor comments that it could be worth addressing to clarify the story being told.

Comments

1. "These reactions include [...] and Hodgkin-Huxley model, and thus are able to generate oscillation." Can the authors be more specific in what they mean by "Hodgkin-Huxley model" here; namely, do they mean a reaction schema equivalent to the Hodgkin-Huxley circuit, or do they mean precisely the ion channel/trans-membrane physics of voltage potentials mediated by potassium ion channels? I assume they mean a model of voltage-gated channels of a different type.

2. Given the complexity of the models and biochemistry involved, the authors only explored a rather small portion of the parameter space, leaving justification of this to [32]. It could be worth providing slightly more justification for the proposed modelling framework and the parameters chosen. In particular, are the concentrations of all substances such that deterministic reaction–diffusion frameworks are appropriate? Brief mention is made of stochastic models, which can exhibit rather different qualitative and quantitative behaviours from the studied models, and may be more appropriate at this sub-cellular scale.

3. Roughly speaking, Section 2.2 shows experimental evidence that Ca^{2+}-cAMP oscillations occur in-phase within the membrane nanodomain, whereas these oscillations are perfectly out-of-phase outside of the nanodomain. Both theoretical models of this, given in Sections 2.1 and 2.3, corroborate these findings. However, is it clear that these theoretical models predict phase differences between perfectly in or out of phase oscillations, without the possibility of phase differences between these extremes? In the model in 2.1, presumably there is not a harsh spatial boundary of the nanodomain, as it is described by a Gaussian. The phase appears to change around $x \approx 50$nm, at least by eye looking at, e.g., Figure 2F, though it is hard to tell how this compares with Figure 2C showing oscillations presumptively inside the nanodomain at $x = 49$nm. Similarly, for a small range of v in Fig 4D, one can also see the possibility of oscillations with inconsistent phase differences (though see comments below on why I think this may

*By signing this review, I waive my right to anonymity.

be erroneous in the Figure). Physically, I presume there is a well-defined boundary of the nanodomain, and that oscillations which do not fit into these two categories are not experimentally accessible. But I wonder if the authors could comment on this, particularly to note if phases in-between exactly in-phase or out-of-phase (or other temporal dynamics) are possible, and if these are observed more readily in other parameter regimes.

4. While equation (2) does not admit a simple closed form solution, I think the authors explanation can be made more simply by directly manipulating this equation. All of the following is very crude/heuristic, but one can make it more rigorous if desired. Firstly, note that if $v = \frac{k_1}{k_2}$, then there is a steady solution given by $x(t) = v$. It is not so trivial to do as the system is nonautonomous, but one can work out that this solution is always stable when it exists (I think). One can also work out a heuristic for why this parameter value is a bifurcation between in or out of phase solutions. Consider the special case that $x(0) = v$, and then one can sign the derivative[1] $x' \approx k_1 - k_2 v$ at $t = 0$ depending on whether $v < \frac{k_1}{k_2}$ or $v > \frac{k_1}{k_2}$. In the former case, one expects in-phase oscillations (as $\sin(t) + 1.1$ is an initially increasing signal), and in the latter case on expects out-of-phase oscillations, at least at this heuristic level. I believe one can see these dynamics play out in Fig 4C, as the flat horizontal line in 4C(ii) looks to be where $x(t) = v = 1$, which is exactly where one expects a steady solution and this bifurcation in phase to occur. In light of this, I'm a bit confused by some of the authors reports of not exactly in-phase or out-of-phase oscillations in Fig 4D (i.e. data points between these two kinds of oscillations). Could these be numerical errors, or depend on the initial data in some way? My intuition is that the initial data shouldn't matter if the analysis is carried out sufficiently far away from transients, but this is worth checking (as the initial conditions were not reported). It would be worth checking these simulation results carefully to ensure they are robust.

5. Related to this, I'm a bit confused by the comment that low values of k_1 and k_2 (Fig 4C(i-ii)) don't exhibit this transition for increasing v, but that high values (Fig 4Ciii) do? I think this may be a question of terminology, as I would say all three of these panels exhibit a change in phase for increasing values of v, in terms of purely where peaks and troughs are. The authors may have a more nuanced definition in mind relative to oscillations around a fixed base value, in which case only Fig 4C(iii) exhibits this transition as clearly. Note that again one can get some heuristic information by considering k_1 and k_2 to be asymptotically large, in which case one expects the dynamics to be an oscillation around the value $x^* = \frac{k_1}{k_2}$, whereas for small k_1 and k_2 values, the oscillations themselves (and hence v) will play a larger role in determining the mean response.

[1] Strictly the inference from this assumption is not valid, and only holds when k_1 and k_2 are asymptotically large relative to $S(t)$.
