# Supplementary material for: Spatiotemporal orchestration of calcium-cAMP oscillations on AKAP/AC nanodomains is governed by an incoherent feedforward loop
Source: PLoS Comput Biol. 2024 Oct 31;20(10):e1012564. doi: 10.1371/journal.pcbi.1012564 (PMC11556706; doi:10.1371/journal.pcbi.1012564)
Supplement: Attachment — Submitted filename: Response_to_reviewers.pdf [file pcbi.1012564.s015.pdf]

Reviewer #1 (Comments to the Author):

Qiao et al study spatial oscillations of Ca^{2+} and cAMP around AKAP/AC nanodomains. They develop a model for these oscillations, point out that it includes an incoherent feedforward component, and explore what happens at different positions from the nanodomain. They also suggest that Turing dynamics may underlie nanodomain formation, and perform simulations on experimentally motivated domain distributions.

Response: We thank the reviewer for their positive and thoughtful assessment of the manuscript. We have now edited the manuscript to address the reviewer's comments.

General comments

1.1. Time delay between Ca^{2+} and cAMP peak. The explanation of why there is a sharp transition in time delay with distance is weak. From Fig 2C, it appears that it is a subtle matter of swapping between two very similar peaks. The normalization makes it unclear how large is the absolute amplitude. In the abstracted model with a sinusoidal approximation, the amplitude of the oscillation drops close to zero at the transition point.

Response: We apologize for the confusion. We agree that the time delay between Ca^{2+} and cAMP is caused by the swapping between two peaks of cAMP, one of which is in-phase with Ca^{2+} and the other is out-of-phase.

We elaborate on the dynamics of Ca^{2+} and cAMP below. When going from the center of the nanodomain to the region far from the nanodomain, the dynamics of cAMP have the following properties: (1) the cAMP dynamics exhibits one in-phase peak with Ca^{2+}, one high in-phase peak and one low out-of-phase peak, one low in-phase peak and one high out-of-phase peak, and finally one out-of-phase peak; (2) in-phase peaks at different locations are achieved at the same time, and so do the out-of-phase peaks; (3) the cAMP level decreases during the process. Combining the properties (1) and (2), the sharp transition of the time delay happens at the location where the in-phase and out-of-phase peaks exhibit the same height, i.e., near 50 nm (see the lower panel in Fig 2D in the revised manuscript).

For the simplified ODE model, when decreasing the activation strength v to mimic the shifting from the center to the edge of the cell membrane, the dynamics of $x(t)$ show the following properties: (1) there is one in-phase peak with the signal $S(t)$, and then one out-of-phase peak with the signal $S(t)$; (2) in-phase peaks for different values of v occurs at the same time, and so do the out-of-phase peaks; (3) the $x(t)$ level decreases during the process. Therefore, based on the properties (1) and (2), there still exists a sharp transition of the time delay in this simplified model, which is consistent with the results in Fig 2C. We acknowledge that although the simplified model can also reproduce the decreased level of cAMP, it fails to capture the two-peak behavior in Fig 2C.

Action taken: In the revision, we have added a new panel C in Fig 2 to show the sharp transition and the non-normalized oscillatory dynamics. To explain the sharp transition in the full model, we edited the text in lines 177-195 in Section "Phase oscillations of Ca^{2+} and cAMP are driven by active AC and PDE". To demonstrate the failure of the simple ODE model to capture the two-peak behavior, we revised lines 316-323 in Section "A simple incoherent feedforward loop explains in-phase oscillation and inversely out-of-phase oscillation".

1.2. The authors frequently use the phrase "perfectly out-of-phase Ca^{2+}/cAMP oscillation". See Figure 2. This is misleading. An out-of-phase signal is generally interpreted to be pi radians away from the in-phase signal. In this case the angular phase delay, and also the time delay are

quite small. The time delay is 20s compared to the oscillation period of 300 s. This is a phase difference of only about 0.4 radians. The reason is that the Ca rises rapidly from valley to peak, and the authors appear to consider the valley to peak phase as perfectly opposite.

Response: In the original manuscript, we used the term "perfectly out-of-phase" to describe the specific alignment where the peak of Ca^{2+} coincides with the valley of cAMP. However, we acknowledge that the term "perfectly out-of-phase" is typically understood to denote a 180-degree phase difference, which does not accurately reflect the situation in our study.

Action taken: To avoid any potential confusion, we have revised the manuscript by replacing "perfectly out-of-phase" with the term "inversely out-of-phase" throughout the main text and figures. Additionally, to ensure clarity, we have provided a definition for "inversely out-of-phase" in the abstract (lines 24-25) and when it first appears in the Results section (lines 223-224), explicitly describing it as the alignment where the peak of Ca^{2+} coincides with the valley of cAMP.

1.3. The authors also frequently refer to incoherent feedback, which arises from the opposite effects of AC and of PDE on cAMP (formation and removal, respectively). My reading of the provided data is that this is not a good characterization of their feedback system. The reason is that AC is present only in the nanodomain, and from what I can see (esp Fig 2), all the relevant dynamics of the oscillatory system reside in the nanodomain. With this interpretation, we have an oscillatory Ca and cAMP signal generated at the nanodomain, with gradually declining levels of cAMP as one goes further away. Ca remains relatively high because of its fast diffusion. cAMP is then in phase with Ca near the nanodomain, but further away the effect of PDE dominates, hence it is lower at the time of the Ca peak. I note that the authors have plotted panel 2C and 2F as normalized cAMP, so one does not get to see that actually cAMP is quite small further away where PDE dominates. As mentioned above, the absolute values should also be reported. My interpretation is easily tested in a non-spatial model with the same reaction scheme. I expect that the behavior will be just like the nanodomain region of the current model. Hence, to summarize the critiques here

Response: We agree with the reviewer that (1) the oscillatory dynamics in the AKAP/AC nanodomain are driven by the excitatory arm, that is, AC favors cAMP synthesis; (2) for the region far from the nanodomain, the excitatory arm disappears and PDE dominates, leading to the degradation of cAMP and thus a quite small cAMP level. Therefore, the observed out-of-phase behavior between Ca^{2+} and cAMP far from the nanodomain is not caused by the excitatory arm. In the original manuscript, we demonstrated that this out-of-phase behavior far from the nanodomain comes from the inhibitory arm (the negative regulation from PDE to cAMP) instead of the excitatory arm. Based on the response below, deleting the inhibitory arm far from the nanodomain abolishes the cAMP oscillation, indicating the important role of the inhibitory arm.

(1) The feedback is not incoherent, it is entirely driven by the excitatory arm of the pathway.

Response: We agree that the oscillation dynamics on the AKAP/AC nanodomain is driven by the excitatory arm of the pathway. However, the out-of-phase behavior far from the nanodomain is not driven by the excitatory arm; it is driven by the inhibitory arm. Furthermore, once we deleted the inhibitory arm and the excitatory arm simultaneously, cAMP concentration cannot oscillate.

Action taken: In the revision, to illustrate the role of the excitatory and inhibitory arms, we ran two new simulations: 1) there is no AKAP/AC nanodomain on the cell membrane, and 2) neither AKAP/AC nanodomain nor PDE exists. The corresponding simulation results are in panel B in new S1 Fig and new S2 Fig, where the absolute values of Ca^{2+}, AC, PDE, cAMP, PKA, and

membrane voltage V are shown. The simulation result of the first case shows that the out-of-phase behavior between Ca^{2+} and cAMP still exists in the absence of the AKAP/AC nanodomain. Note that the absence of AKAP/AC nanodomain also means the deletion of the excitatory arm, since the excitatory arm requires AC. Thus, this result indicates that the out-of-phase behavior is not driven by the excitatory arm, and is likely driven by the the inhibitory arm. As for the second case, the Ca^{2+} can still oscillate while cAMP cannot; this was also found in the experiment when cells were treated with the PDE inhibitor 3-isobutyl-1-methylxanthine (IBMX) (Figure 5E in (*1*)). Thus, the simulation result in the second case indicates that the inhibitory arm is important for cAMP oscillation. Taken together, the inhibitory arm is key to cAMP oscillation far from the nanodomain, and the excitatory arm drives the in-phase behavior on the nanodomain. The above analysis was in lines 250-262 in the revised manuscript.

(2) The oscillatory dynamics are driven by the excitatory arm in the nanodomain and this can be tested using a single-compartment model to replicate the nanodomain properties.

Response: As we mentioned in the response to comment (1), we ran two new simulations where the excitatory arm is deleted and both arms are deleted. These new simulations still have two compartments, that is, membrane and cytosol, which allow for the bulk-surface reactions. The simulation results indicate that the excitatory arm contributes to the in-phase behavior rather than the out-of-phase behavior, and that the inhibitory arm contributes to the cAMP oscillation.

Action taken: We added S1B Fig, S2 Fig and text in lines 250-262 in the revised manuscript to show and analyze the new simulation results.

(3) Any substantial inhibitory effect of PDE on cAMP occurs far from the nanodomain, hence the PDE negative feedback effect on cAMP has little effect on the total PKA activity.

Response: We agree that the negative effect of PDE on cAMP has little effect on the total PKA activity. However, due to the slow diffusion of PKA (D_{PKA}=0.01 $\mu m^2\,s^{-1}$), PDE can affect the cAMP and the subsequent PKA concentration locally, causing a spatial heterogeneity of PKA activity (see panel A(vi) in S1 Fig). This spatial heterogeneity might be crucial for cells to transmit signals (*2*). Furthermore, the out-of-phase Ca^{2+}/cAMP oscillation seems to be more ubiquitous than in-phase Ca^{2+}/cAMP oscillation according to Figure 2C in (*3*) and Figure 5 in (*1*).

Action taken: In the revision, to show the low PKA activity outside the nanodomain, we have added a new panel A in S1 Fig. In this panel, the cAMP level is higher on the AKAP/AC nanodomain than that far from the nanodomain, and so is the PKA level. This result suggests that although the negative regulation from PDE to cAMP has little impact on the total PKA activity, this regulation contributes to the low PKA level far from the nanodomain. To introduce the spatial heterogeneity of PKA in the model, lines 162-164 were edited in Section "Phase oscillations of Ca^{2+} and cAMP are driven by active AC and PDE". To discuss the possible biological functions of out-of-phase behavior, lines 509-512 were also revised in Discussion section.

(4) The cAMP levels in the region where PDE dominates are very small and unlikely to modulate PKA significantly.

Response: We agree that the cAMP levels in the region where PDE dominates are very small. Thus, the PKA level is also small in this region. As we mentioned in the response to comment (3), the small PKA level in this region might be important for signal transduction, and the behavior far from nanodomain might contribute more to the entire Ca^{2+}/cAMP behavior because experimental

data in (*3*) and (*1*) both show an out-of-phase behavior between Ca^{2+} and cAMP.

Action taken: We added a new panel A in S1 Fig to show the absolute value of cAMP and PKA, and corresponding explanations were also added in lines 162-164 to introduce the low value of PKA. To discuss the possible biological functions of out-of-phase behavior, lines 509-512 were edited in Discussion section.

1.4. The authors then fold in a somewhat orthogonal question about formation of nanodomains. They refer to STORM data, smooth it, and obtain small peaks which they interpret as naondomains. Unfortunately they have used one sample, which is not a reliable basis for interpreting nanodomain presence. Further, they choose a Gaussian smoothing function of 60 nm without a clear rationale for this length scale.

Response: We thank the reviewer for these insightful remarks. We undertook the following steps to address these comments. In the revision, we searched the literature for the diffusion coefficient of AC and then performed a new analysis of adenylyl cyclase (AC) STORM data. The low diffusion coefficient of AC and near 42% immobile fraction indicate that AC nanodomain is quite stable. As we elaborate below, these AC nanodomains may not exhibit obvious periodicity from our new analysis.

Action taken: For the comment "they choose a Gaussian smoothing function of 60 nm without a clear rationale for this length scale.", we ran a new simulation with a Gaussian smoothing function whose FWHM is 30 nm and added a new S5 Fig to show the simulation results. In the new S5 Fig, the in-phase and out-of-phase Ca^{2+}/cAMP oscillations also occur. This is consistent with that lowering AC level leads to the emergence of out-of-phase Ca^{2+}/cAMP oscillation outside the nanodomain (Fig 8B-D), because the AC activity with a 30 nm FWHM is less than or equal to that with 60 nm FWHM at any location on the top surface. To illustrate this, in the revision, we added sentences in lines 469-472 in Section "Prediction of the time delay from a realistic distribution of AKAP/AC nanodomains".

(1) Can the authors do the analysis in a statistically thorough manner, with multiple samples and multiple biological replicates? This would be the starting point for any interpretation on periodicity of the nanodomains, which is a requisite to interpret them as Turing patterns.

Response: In the revision, we analyzed three samples of AC STORM images and added a new S4 Fig to demonstrate that AC nanodomains are not periodic. The region we analyzed is $10\mu m \times 10\mu m$ (middle panels in new S4 Fig). For each region, the mean-shift algorithm was utilized to determine the clusters, where the center of each cluster is the center of the red circle in middle panels. The radius of the red circle is defined to make sure that 95% percent of cluster members are in the circle. Panels (iii) and (iv) in the new S4 Fig show the distribution of nearest-neighbor distance and cluster size (or radius), respectively. It can be seen that the nearest-neighbor distance and cluster size are both diverse, indicating that AC nanodomains are not periodic. Thus, the Turing pattern is just an idealized distribution of AC nanodomains for purposes of analyses in our model.

Action taken: In the revision, we changed the section title "AKAP/AC nanodomain formation can be explained by Turing patterns" to "Prediction of the time delay from idealized Turing pattern-based AKAP/AC nanodomains", added a new S4 Fig, and revised the text in lines 391-399.

(2) On the basis of this, can they obtain the wavelength of the proposed Turing pattern?

Response: According to panels (iii) in the new S4 Fig, the mean wavelength (or the nearest-

neighbor distance) of the AC nanodomain is 341 nm with three samples. This value is larger than that in Figure 3b in (*4*) (292 nm), which may be due to parameter settings in the mean-shift algorithm or the artificial variations caused by the staining of STORM data.

Action taken: In the revision, to discuss the difference of nanodomain distance in the experiment and model, we added text in lines 578-584 in Discussion section.

(3) Further, can they use a larger sample set to obtain a population estimate of proposed nanodomain size?

Response: According to panels (iv) in the new S4 Fig, the mean AC nanodomain size is 105 nm with three samples. This value is close to that reported in Figure 3b in (*4*) (88 nm).

Action taken: In the revision, the comparison of nanodomain size in the experiment and model was also added in lines 578-584 (See response to comment (2)).

(4) I was looking to see an analysis that justified the strong assumption that nanodomains were indeed formed through a Turing mechanism. My reading is that the authors have come up with a potential Turing-like mechanism for nanodomain formations, but I do not see experimental support for either the existence of this mechanism nor the existence of Turing like regular patterns. Hence at this stage this appears like a hypothesis, which could be subject to future testing.

Response: We agree with the reviewer.

Action taken: In the revision, to acknowledge that reactions in the Turing model lack experimental evidence, we revised lines 409-412.

(5) Can the authors provide evidence that the AC8 distributions reported are stable, that is, that these are long-lived nanodomains rather than transients or experimental noise?

Response: We searched the literature (*4*) for the diffusion property of AC. Although STORM data provides the location of each AC molecule, it cannot be obtained continuously because the cell is fixed when collecting the STORM image. Therefore, it is hard to determine how long the AC nanodomain will persist. However, the mobility of AC molecules can be measured by fluorescence recovery after photobleaching (FRAP) experiment. By performing FRAP, Tenner et al. revealed that AC diffuses slowly with the diffusion coefficient of 0.019 ± 0.002 $\mu m^2/s$ and exhibits a significant immobile fraction (near 42.3%) (Figure 3—figure supplement 3 in (*4*)). Therefore, AC nanodomains are not transient compared with the time scale studied in the manuscript.

Action taken: In the revision, we added the sentences in lines 395-397 to illustrate the long-lived property of AC nanodomains.

(6)There are potentially interesting interactions here between adjacent nanodomains (if they are verified), and dependencies on their spacing. These are explored briefly in Figure 7. However the current single-sample analysis does not support such generalization.

Response: We agree with the reviewer.

Action taken: To clarify the limitation of our study, in the revision, we revised sentences in lines 584-589 in the last paragraph of Section Discussion.

1.5. The authors state "These reactions include negative feedback, incoherent feedback loop, and Hodgkin-Huxley model, and thus are able to generate oscillation." The equation form for K channels

is not in the HH form. Also I do not see an equation for computing V, nor do I see plots for V in the figures.

Response: Sorry for the confusion. We agreed that the equations are not in the Hodgkin-Huxley form. In the original manuscript, we used a modified version of the Chay-Keizer model to describe the dynamics of membrane voltage (*3–6*), which was the first and second equations on the cell membrane Γ in the equation (S1) in the original Supplement. Specifically, we considered four ion channels: Ca^{2+} gated K^+, Ca^{2+}, K^+, and leak channels. Then, similar to the Hodgkin-Huxley framework, the current flowing through the membrane, i.e., $C_m \frac{dV}{dt}$, was the negative of the sum of currents passing through the ion channels. Here, C_m and V are membrane conductance and voltage.

Action taken: In the revision, to clearly introduce the voltage model we used, we have added a voltage module in Fig 1C, revised the lines 136-142 in Section "Phase oscillations of Ca^{2+} and cAMP are driven by active AC and PDE ", and added new Methods subsections in lines 676-699.

Minor points

1.6. Pg 52-53, table S3: "Voltage of K^+ channel" is incorrect. I think the authors mean to say reversal potential of K^+ channel. Similarly for Ca^{2+} channel.

Response: Sorry for the mistake.

Action taken: In the revision, we have corrected the explanations of E_{KCa}, E_{Ca}, E_K, and E_L in S4 Table (Table S3 in the original manuscript) as the reviewer suggested.

Reviewer #2 (Comments to the Author):

Spatiotemporal orchestration of Ca2+-cAMP oscillations on AKAP/AC nanodomains is governed by an incoherent feedforward loop" (PCOMPBIOL-D-24-00398) Overall, the manuscript submitted by Dr. Padmini Rangamani and colleagues is well-written and of general interest to the calcium signaling/cAMP signaling community. The key incoherent circuit that can explain the changing of phase between Ca2+ and cAMP oscillations is a notable finding from the computational work. The key questions posed by the study are answered computationally.

Response: We thank the reviewer's insightful evaluation of the manuscript. We have now edited the manuscript to address the reviewer's comments.

Major comments:

2.1. Ref. 32 appears to have important data for the present study, but is only published as a presentation abstract. It seems that the citation for [32] is incorrect and should refer to `https://elifesciences.org/articles/55013`?

Response: We thank the reviewer for bringing this error to our attention.

Action taken: In the revision, we have corrected the citation to Tenner et. al., eLife, 2020.

2.2. Additionally, the novelty of the present computational work should be more expansively delineated from the eLife paper. It was not clear what is meant by "perfect property" of the out-of-phase behavior, as this out-of-phase activity seems to be discussed in the 2020 paper. The two studies should be explicitly compared and differentiated. Example: "have been experimentally well studied in [32],"

Response: The novelty of this computational work is that we revealed the out-of-phase behavior described in the eLife paper is not an arbitrary phase difference: Ca^{2+} reaches its trough at the same time when cAMP reaches the peak. Based on this discovery, we further identified the underlying mechanism as an incoherent feedforward loop, and investigated how this oscillation behavior is affected by biophysical proprieties of cells and nanodomain distributions. In the original manuscript, to emphasize that the out-of-phase Ca^{2+}/cAMP oscillation exhibits the same Ca^{2+} trough time and cAMP peak time, we used the "perfectly out-of-phase". However, the term "perfectly" is misleading since it is typically understood as a 180-degree phase difference, which is not the case in our study (also see the response to the comment 1.2 from Reviewer 1).

In the original manuscript, we wanted to use the sentence "Since the cAMP dynamics at the cell membrane have been experimentally well studied in [32]" to explain why we focused only on simulation results at the cell membrane rather than in the cytosol. However, this caused confusion that this computational work lacks novelty compared to the work by Tenner et al.

Action taken: To avoid the confusion, we have revised the manuscript by replacing "perfectly out-of-phase" with the term "inversely out-of-phase" throughout the main text and figures. Additionally, we added the definition of "inversely out-of-phase" in the abstract (lines 24-26) and the time when it first appears in the Results section (lines 223-224).

As for the confusion caused by "have been experimentally well studied in [32]," we have rewritten this sentence in lines 166-168.

2.3. Can specific downstream biological functions be assigned to the in-phase and out-of-phase behavior inside and outside the domains?

Response: We thank the reviewer for this suggestion. The direct validation of downstream biological functions of in-phase and out-of-phase Ca^{2+}/cAMP oscillations in pancreatic β cells is not yet fully understood. However, according to published studies, the in-phase Ca^{2+}/cAMP oscillation on the AKAP/AC nanodomain may regulate the insulin secretion by AKAP because of the anchoring property of AKAP. AKAP can anchor both PKA and guanine exchange factor Epac, and PKA and Epac are regulated by cAMP. Both PKA and Epac play important roles in insulin secretion: these two types of molecules not only improve the mobilization of secretory granules (*7–9*) but also interact with proteins involved in the exocytosis process such as SNAP-25 and Snapin (*10–13*). On the other hand, the out-of-phase Ca^{2+}/cAMP oscillation encodes the information in extracellular signals (*1*).

Action taken: In the revision, we added the role of the out-of-phase Ca^{2+}/cAMP oscillation in lines 509-512 in the Discussion section. These lines together with text in lines 499-509 discuss the possible role of Ca^{2+}/cAMP phase behavior.

2.4. It would be helpful to provide a more specific rationale within the text for model parameters and calibration to save the reader time in referring to the previous studies.

Response: We thank the reviewer for the good suggestion.

Action taken: In the revision, to provide the justification for parameters, we have not only added the source of kinetic parameters in S4 Table but also added the sentences in lines 143-150 and 156-164 in Section “Phase oscillations of Ca^{2+} and cAMP are driven by active AC and PDE”.

Minor issues or unclear points:

2.5. Mention the specific factors in this summary statement: “but some of these factors can affect the time delay for the perfectly out-of-phase Ca2+/cAMP oscillation.”

Response: We thank the reviewer for this suggestion.

Action taken: In the revision, we rewrote this sentence as follows: ”but cellular surface-to-volume ratio and compartment size can affect the time delay for the perfectly out-of-phase Ca^{2+}/cAMP oscillation while the distance between two nanodomains cannot.”

2.6. The role of compartment height needs to be clearly explained in the text.

Response: The compartment height can be interpreted as the cell volume in the neighborhood of the domain.

Action taken: In the revision, to give predictions about the role of compartment height, we revised lines 385-387 in Section ”Cellular compartment size determines the time delay for the perfectly out-of-phase Ca^{2+}-cAMP oscillation”.

2.7. The term compartment size was somewhat confusing in relation to the biological structure and organization of the cell. Clarity in terminology would be helpful.

Response: The compartment size is the volume of the simulation domain. It reflects the cell volume to some extent. The role of cell shape and volume has been widely explored in different cell

types (*14–16*), and thus we expect that different compartment sizes can regulate cellular signaling.

Action taken: In the revision, to explain the biological meaning of compartment height and width, we edited text in lines 368-371 in the Section "Cellular compartment size determines the time delay for the perfectly out-of-phase Ca^{2+}-cAMP oscillation".

Reviewer #3 (Comments to the Author):

The authors explore models of Ca2+-cAMP oscillations and how they are influenced by the presence of AKAP/AC nanodomains which form on cell membranes. They argue that these oscillations play a pivotal role in many diseases and developmental/physiological function, and that the impact of membrane nanodomains on the local profiles of such oscillations can have important effects. They use a range of simple and quite complicated models of reaction-diffusion type to explore in-phase and perfectly out-of-phase oscillations mediated by being inside and outside, respectively, of these nanodomains. They show that such dynamics are qualitatively robust to biophysical details, such as compartment size and geometry/spacing of nanodomains. They also postulate a simple model of nanodomain formation via a Turing reaction-diffusion mechanism, though caveat this as only one plausible mechanism for such pattern formation. Importantly, they highlight throughout a simplistic motif of a feedforward incoherent feedback loop that can help explain the phase of these Ca2+-cAMP oscillations. Overall I find the paper interesting, and broadly well-written. Below are a few rather minor comments that it could be worth addressing to clarify the story being told.

Response: We thank the reviewer's positive assessment of the manuscript. We have now revised the manuscript to address the reviewer's comments.

3.1. "These reactions include [...] and Hodgkin-Huxley model, and thus are able to generate oscillation." Can the authors be more specific in what they mean by "Hodgkin-Huxley model" here; namely, do they mean a reaction schema equivalent to the Hodgkin-Huxley circuit, or do they mean precisely the ion channel/trans-membrane physics of voltage potentials mediated by potassium ion channels? I assume they mean a model of voltage-gated channels of a different type.

Response: In the original manuscript, the framework for the voltage model is based on Hodgkin-Huxley model, but the exact form follows a modified version of the Chay-Keizer model (*3–6*). The cell membrane voltage V is mediated by a K^+ (potassium) channel, a Ca^{2+} gated K^+ channel, a Ca^{2+} channel, and a leak channel. The currents of these four ion channels are denoted as I_K, I_{KCa}, I_{Ca}, and I_L. Then, the time evolution of the membrane voltage V is modeled by $\frac{1}{C_m}(-I_K - I_{KCa} - I_{Ca} - I_L) + D_V \nabla_\Gamma^2 V$, where C_m is the capacitance of cell membrane and D_V is the diffusion coefficient of the membrane voltage (also see the response to the comment 1.5 from Reviewer 1).

Action taken: In the revision, to introduce the voltage model we used, we have added a voltage module in Fig 1C, rewritten the lines 136-142 in Section "Phase oscillations of Ca^{2+} and cAMP are driven by active AC and PDE ", and added new Methods subsections in lines 676-699.

3.2. Given the complexity of the models and biochemistry involved, the authors only explored a rather small portion of the parameter space, leaving justification of this to [32]. It could be worth providing slightly more justification for the proposed modelling framework and the parameters chosen. In particular, are the concentrations of all substances such that deterministic reaction–diffusion frameworks are appropriate? Brief mention is made of stochastic models, which can exhibit rather different qualitative and quantitative behaviours from the studied models, and may be more appropriate at this sub-cellular scale.

Response: Thanks for the good suggestion.

Action taken: In the revision, to provide the justification of kinetic parameter settings, we have not only added the source of kinetic parameters in S4 Table but also added the sentences in lines 143-150 and 156-164 in Section "Phase oscillations of Ca^{2+} and cAMP are driven by active AC and

PDE".

Moreover, to demonstrate the model limitation in not considering the effect of stochasticity, in the revision, we edited lines 593-597 in the last paragraph of the Discussion section.

3.3. Roughly speaking, Section 2.2 shows experimental evidence that Ca2+-cAMP oscillations occur in-phase within the membrane nanodomain, whereas these oscillations are perfectly out-of-phase outside of the nanodomain. Both theoretical models of this, given in Sections 2.1 and 2.3, corroborate these findings. However, is it clear that these theoretical models predict phase differences between perfectly in or out of phase oscillations, without the possibility of phase differences between these extremes? In the model in 2.1, presumably there is not a harsh spatial boundary of the nanodomain, as it is described by a Gaussian. The phase appears to change around $x \approx$ 50nm, at least by eye looking at, e.g., Figure 2F, though it is hard to tell how this compares with Figure 2C showing oscillations presumptively inside the nanodomain at $x =$ 49nm. Similarly, for a small range of v in Fig 4D, one can also see the possibility of oscillations with inconsistent phase differences (though see comments below on why I think this may be erroneous in the Figure). Physically, I presume there is a well-defined boundary of the nanodomain, and that oscillations which do not fit into these two categories are not experimentally accessible. But I wonder if the authors could comment on this, particularly to note if phases in-between exactly in-phase or out-of-phase (or other temporal dynamics) are possible, and if these are observed more readily in other parameter regimes.

Response: We thank the author for this suggestion. For the 3D reaction-diffusion model in Fig 1C, there is no sudden change for AKAP and AC on the membrane, since the initial condition of AKAP and AC are modeled by Gaussian distribution. In addition to the in-phase and out-of-phase behavior, the phase difference during the transition of these two behaviors is as follows: the cAMP dynamics shows two peaks near the boundary of the AKAP/AC nanodomain, one of which is in phase with Ca^{2+} and the other is out of phase with Ca^{2+}. Furthermore, the in-phase peaks of cAMP occur at the same time, and so do the out-of-phase peaks of cAMP.

For the simple ODE model, we did not observe any other type of peaks except the ones in Fig 4, which is also validated by theoretical analysis (see response to the next comment).

Action taken: To illustrate this oscillatory behavior near the nanodomain boundary, in the revision, we added a new panel C in Fig 2 to show the dynamics of Ca^{2+} and cAMP on the nanodomain, near the edge of the nanodomain, and far from the nanodomain. The related main text was also revised in lines 177-195.

At the same time, to discuss the failure of the simple ODE model to capture the two-peak behavior, we revised the text in lines 316-323.

3.4. While equation (2) does not admit a simple closed form solution, I think the authors explanation can be made more simply by directly manipulating this equation. All of the following is very crude/heuristic, but one can make it more rigorous if desired. Firstly, note that if $v = \frac{k1}{k2}$, then there is a steady solution given by $x(t) = v$. It is not so trivial to do as the system is nonautonomous, but one can work out that this solution is always stable when it exists (I think). One can also work out a heuristic for why this parameter value is a bifurcation between in or out of phase solutions. Consider the special case that $x(0) = v$, and then one can sign the derivative (Strictly the inference from this assumption is not valid, and only holds when k_1 and k_2 are asymptotically large relative to $S(t)$.) $\frac{dx}{dt} \approx k_1 - k_2 v$ at $t = 0$ depending on whether $v < \frac{k1}{k2}$ or $v > \frac{k1}{k2}$. In the former case, one expects in-phase oscillations (as $\sin(t) + 1.1$ is an initially increasing signal), and in the latter case on expects out-of-phase oscillations, at least at this heuristic level. I believe one can see these dynamics play out in Fig 4C, as the flat horizontal line in 4C(ii) looks to be where $x(t) = v = 1$,

which is exactly where one expects a steady solution and this bifurcation in phase to occur. In light of this, I'm a bit confused by some of the authors reports of not exactly in-phase or out-of-phase oscillations in Fig 4D (i.e. data points between these two kinds of oscillations). Could these be numerical errors, or depend on the initial data in some way? My intuition is that the initial data shouldn't matter if the analysis is carried out sufficiently far away from transients, but this is worth checking (as the initial conditions were not reported). It would be worth checking these simulations results carefully to ensure they are robust.

Response: We thank the reviewer for the very good suggestion. In the revision, by implementing this idea, we proved that there are only two phase differences when changing the activation strength v, and that the transition occurs at $v = \frac{k_1}{k_2}$. This means that the data points between exactly in-phase and out-of-phase oscillations in the original manuscript come from numerical errors.

Action taken: To illustrate the fact that there are only two types of time delay when varying v in Fig 4D, we made two modifications in the revision:

(1) to briefly explain why there are only two phase differences with varied v, we edited sentences in lines 301-303 and 333-340 in the main text. We also added the detailed analysis is in lines 710-724 in the Methods section.

(2) We modified Fig 4D by combining the fact that the transition occurs at $v = \frac{k_1}{k_2}$. We double-checked the data points near the transition, and found that the corresponding dynamics of $x(t)$ oscillates with very small amplitude near the constant $\frac{k_1}{k_2}$ (see the following figure). At the same time, the numerical results did not show a regular wave no matter how we changed the time step or ODE solver. Thus, the peak time of $x(t)$ is not accurate. In the revision, we deleted the data points in Fig 4D whose y axis value is neither in the upper line nor in the bottom line, and then plotted $v = \frac{k_1}{k_2}$ as the transition point. The caption of Fig 4D was also revised to reflect how to plot the transition curve.

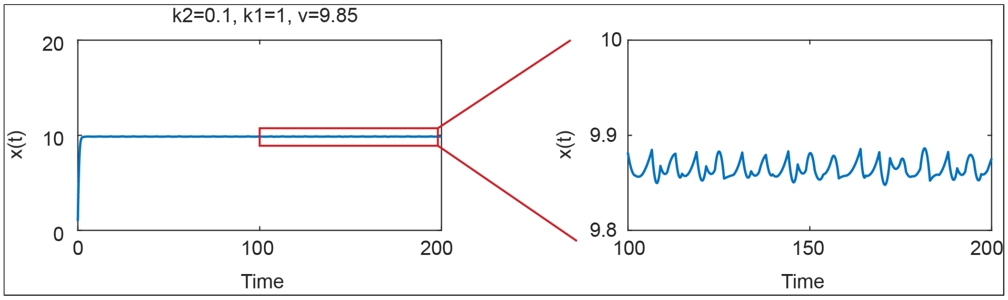

3.5. Related to this, I'm a bit confused by the comment that low values of k_1 and k_2 (Fig 4C(i-ii)) don't exhibit this transition for increasing v, but that high values (Fig 4Ciii) do? I think this may be a question of terminology, as I would say all three of these panels exhibit a change in phase for increasing values of v, in terms of purely where peaks and troughs are. The authors may have a more nuanced definition in mind relative to oscillations around a fixed base value, in which case only Fig 4C(iii) exhibits this transition as clearly. Note that again one can get some heuristic information by considering k_1 and k_2 to be asymptotically large, in which case one expects the dynamics to be an oscillation around the value $x^* = \frac{k1}{k2}$, whereas for small k_1 and k_2 values, the oscillations themselves (and hence v) will play a larger role in determining the mean response.

Response: After analyzing the solution of $x(t)$ around the $\frac{k_1}{k_2}$ (see the response to the comment 3.4), we are also able to explain why the case with large k_2 exhibits the transition from perfectly out-of-phase to in-phase behavior.

Action taken: In the revision, we added the brief analysis in lines 340-344. The detailed analysis was added in lines 724-732.

References

1. L. R. Landa Jr. *et al.*, Interplay of Ca_{2+} and cAMP Signaling in the Insulin-secreting MIN6 beta Cell Line. *Journal of Biological Chemistry* **280**, 31294–31302, ISSN: 0021-9258, DOI `10.1074/jbc.M505657200`, (`https://doi.org/10.1074/jbc.M505657200`) (Sept. 2005).
2. K. Lefkimmiatis, M. Zaccolo, cAMP signaling in subcellular compartments. *Pharmacology and Therapeutics* **143**, 295–304, ISSN: 0163-7258, DOI `https://doi.org/10.1016/j.pharmthera.2014.03.008`, (`https://www.sciencedirect.com/science/article/pii/S0163725814000692`) (2014).
3. Q. Ni *et al.*, Signaling diversity of PKA achieved via a Ca2+-cAMP-PKA oscillatory circuit. *Nature Chemical Biology* **7**, 34–40, ISSN: 1552-4469, DOI `10.1038/nchembio.478`, (`https://doi.org/10.1038/nchembio.478`) (Jan. 2011).
4. B. Tenner *et al.*, Spatially compartmentalized phase regulation of a Ca^{2+}-cAMP-PKA oscillatory circuit. *eLife* **9**, ed. by S. Mayor, J. A. Cooper, A. Nadler, e55013, ISSN: 2050-084X, DOI `10.7554/eLife.55013`, (`https://doi.org/10.7554/eLife.55013`) (Nov. 2020).
5. T. R. Chay, J. Keizer, Minimal model for membrane oscillations in the pancreatic beta-cell. *Biophysical Journal* **42**, 181–189, ISSN: 0006-3495, DOI `10.1016/S0006-3495(83)84384-7`, (`https://doi.org/10.1016/S0006-3495(83)84384-7`) (May 1983).
6. C. Fall, E. Marland, J. Wagner, J. Tyson, *Computational Cell Biology* (Springer New York, 2007), ISBN: 9780387224596, (`https://books.google.com/books?id=AdCTvbOzRywC`).
7. T. Shibasaki *et al.*, Essential role of Epac2/Rap1 signaling in regulation of insulin granule dynamics by cAMP. *Proceedings of the National Academy of Sciences* **104**, 19333–19338, DOI `10.1073/pnas.0707054104`, eprint: `https://www.pnas.org/doi/pdf/10.1073/pnas.0707054104`, (`https://www.pnas.org/doi/abs/10.1073/pnas.0707054104`) (2007).
8. E. Renström, L. Eliasson, P. Rorsman, Protein kinase A-dependent and-independent stimulation of exocytosis by cAMP in mouse pancreatic B-cells. *The Journal of physiology* **502**, 105 (1997).
9. L. Eliasson *et al.*, SUR1 Regulates PKA-independent cAMP-induced Granule Priming in Mouse Pancreatic B-cells. *Journal of General Physiology* **121**, 181–197, ISSN: 0022-1295, DOI `10.1085/jgp.20028707`, eprint: `https://rupress.org/jgp/article-pdf/121/3/181/1778193/jgp1213181.pdf`, (`https://doi.org/10.1085/jgp.20028707`) (Feb. 2003).
10. W.-J. Song *et al.*, Snapin Mediates Incretin Action and Augments Glucose-Dependent Insulin Secretion. *Cell Metabolism* **13**, 308–319, ISSN: 1550-4131, DOI `10.1016/j.cmet.2011.02.002`, (`https://doi.org/10.1016/j.cmet.2011.02.002`) (Mar. 2011).
11. J. Vikman *et al.*, Truncation of SNAP-25 reduces the stimulatory action of cAMP on rapid exocytosis in insulin-secreting cells. *American Journal of Physiology-Endocrinology and Metabolism* **297**, PMID: 19509185, E452–E461, DOI `10.1152/ajpendo.90585.2008`, eprint: `https://doi.org/10.1152/ajpendo.90585.2008`, (`https://doi.org/10.1152/ajpendo.90585.2008`) (2009).

12. Q.-F. Wan *et al.*, Protein Kinase Activation Increases Insulin Secretion by Sensitizing the Secretory Machinery to Ca2+. *Journal of General Physiology* **124**, 653–662, ISSN: 0022-1295, DOI 10.1085/jgp.200409082, eprint: https://rupress.org/jgp/article-pdf/124/6/653/1908449/jgp1246653.pdf, (https://doi.org/10.1085/jgp.200409082) (Nov. 2004).

13. A. Tengholm, E. Gylfe, cAMP signalling in insulin and glucagon secretion. *Diabetes, Obesity and Metabolism* **19**, 42–53, DOI https://doi.org/10.1111/dom.12993, eprint: https://dom-pubs.onlinelibrary.wiley.com/doi/pdf/10.1111/dom.12993, (https://dom-pubs.onlinelibrary.wiley.com/doi/abs/10.1111/dom.12993) (2017).

14. P. Rangamani *et al.*, Decoding information in cell shape. *Cell* **154**, 1356–1369 (2013).

15. M. Schmick, P. I. Bastiaens, The Interdependence of Membrane Shape and Cellular Signal Processing. *Cell* **156**, 1132–1138, ISSN: 0092-8674, DOI 10.1016/j.cell.2014.02.007, (https://doi.org/10.1016/j.cell.2014.02.007) (Mar. 2014).

16. A. Haupt, N. Minc, How cells sense their own shape – mechanisms to probe cell geometry and their implications in cellular organization and function. *Journal of Cell Science* **131**, jcs214015, ISSN: 0021-9533, DOI 10.1242/jcs.214015, eprint: https://journals.biologists.com/jcs/article-pdf/131/6/jcs214015/3505011/jcs214015.pdf, (https://doi.org/10.1242/jcs.214015) (Mar. 2018).
